# Greater alteration of gut microbiota occurs in childhood obesity than in adulthood obesity

**DOI:** 10.3389/fped.2023.1087401

**Published:** 2023-01-26

**Authors:** Zhongjia Yu, Xiang-Fang Yu, Xiu Zhao, Zhe Su, Pei-Gen Ren

**Affiliations:** ^1^ Center for Energy Metabolism and Reproduction, Shenzhen Institute of Advanced Technology, Chinese Academy of Sciences, Shenzhen, China; ^2^ Shenzhen College of Advanced Technology, University of Chinese Academy of Sciences, Shenzhen, China; ^3^ Department of Endocrinology, Shenzhen Children's Hospital, Shenzhen, China

**Keywords:** children, adults, gut microbiota, obesity, data mining

## Abstract

The children's gut microbiota, associated with the development of obesity, is in maturation. The impact of obesity on the gut microbiota in childhood could have a more significant effect than on adulthood and eventually be lifelong lasting, but it has been rarely studied. Aimed to discover the difference in gut microbiota between children and adults with obesity, we collected published amplicon sequencing data from National Center for Biotechnology Information (NCBI) and re-analyzed them using a uniform bioinformatic pipeline, as well as predicted the obesity using gut microbiota based on the random forest model. Summarizing common points among these cohorts, we found that the gut microbiota had a significant difference between children with and without obesity, but this difference was not observed in adult cohorts. Based on the random forest model, it was more challenging to predict childhood obesity using gut microbiota than adulthood obesity. Our results suggest that gut microbiota in childhood is more easily affected than in adulthood. Early intervention for childhood obesity is essential to improve children's health and lifelong gut microbiota-related health.

## Introduction

The association between gut microbiota and obesity has been widely discussed in recent decades. However, the etiologic relationship still lacks solid evidence for in-depth analyses (1). Relevant studies focusing on both adulthood and childhood obesity have revealed that the shifts of gut microbiota happen during the development of obesity and during the intervention for obesity (2–5). In adults, the signature of gut microbiota reflects the characteristics of the population in subgroups, including diet preference, living style, and risk factors for metabolic disorders (6). Therefore, the signature of gut microbiota has become one of the most promising factors in predicting metabolic disorders, including obesity. Unlike adults, the gut microbiota of children is maturing; thus, the factors and status of childhood obesity may affect the shaping of gut microbiota and even have a lifelong impact (7). However, minimal studies have been focused on the difference in gut microbiota between adulthood and childhood obesity. Moreover, cohorts from different countries or areas have unique gut microbiota signatures due to genetic factors, diet preferences, and environment. These variants add difficulties in patterning gut microbiota shifts in the development of obesity worldwide. A study reviewing worldwide data to illustrate the different gut microbiota signatures in different countries would add value to understanding the relationship between obesity and gut microbiota. In the present study, we analyzed worldwide raw 16S rRNA amplicon sequencing data of gut microbiota in both adulthood and childhood obesity studies from the National Center for Biotechnology Information (NCBI) database to reveal different traits between adults and children with obesity in the world.

## Materials and methods

### Data resource

All raw data was obtained in the search with keywords “obesity” OR “obese” AND “gut microbiota” OR “microbiome” OR “microbiota” in the BioProject of NCBI. The data released before 1st February 2022 were searched. Only anthropic studies containing at least four healthy individuals were further analyzed. Raw data of 16S amplicon sequencing for each Biosample was collected. Moreover, if the study is about intervention, only basal data of both control and obese individuals were included.

All information on each BioProject was summarized using both published sample sheets (Supplementary Table) and relevant publications. The category of body weight was in line with the sheets. The following rules were used for BMI grouping if it was not mentioned. For children from Non-US and US, the bodyweight category was checked using BMI-for-age check forms from World Health Organization and the Centers for Disease Control and Prevention, respectively. For adults, overweight is BMI between 24.9–30 kg/cm^2^, and obesity is BMI over 30 kg/cm^2^. For the dataset that contains the category information, it was adopted directly in our analyses under the redefinition as the obesity included “overweight”, “obese,” and “superobese” from the BMI category, and other levels were counted as healthy control.

### Data process

Raw data was analyzed using the QIIME2 (version 2021.08) software pipeline (8). Reads were demultiplexed with q2-demux. Then, the DADA2 plugin was implemented for the quality control process, and all phiX reads and chimeric sequences were filtered. Based on the demux summary, single-end sequences were truncated to a length with 160–200 bases (details in Supplementary Table). After denoising, the data using the DADA2 denoise-paired method, representative sequences of each sample were retained. ASVs were filtered out non-bacterial sequences against Silva 99% using method vsearch, respectively. Then, Naive Bayes classifiers pre-trained on Silva 138 99% OTUs full-length sequences were used correspondingly for taxonomy assignment. Afterwards, the number of 16S rRNA copies of each taxon were normalized using a plug-in commend.

### Statistic analyses and visualization

The principal coordinate analysis (PCoA), PERMANOVA test, and visualization were performed using the ImageGP online tool (9). Alpha diversity index was calculated using QIIME2 plug-in commends and visualization using GraphPad. Differences in the abundance of each taxon between healthy and obese individuals were analyzed using DESeq (10). The random sampling for matched sample size and PERMANOVA were tested using R packages (9). For the random forest model test using the R library (randomForest), we tested dataset from each cohorts and merged datasets, which were combined based on taxa levels for children and adults cohorts respectively, after training the model with a 70% sample size (11).

## Results

A total of ten cohorts from nine BioProjects were included, four for childhood obesity (PRJNA647465 (11), PRJNA317290, PRJNA433269 (12), PRJNA637782 (13)) and five for adulthood obesity (PRJNA483803, PRJNA339677 (14), PRJNA631293 (15), PRJNA273824, PRJNA417691 (16)). Among these projects, 601 samples of raw data met our categories and were further analyzed, and the detail of each BioProject is shown in Supplementary Tables S2–S11. To our knowledge, all the bioproject used stool samples for the amplicon sequencing.

**Figure 1:**
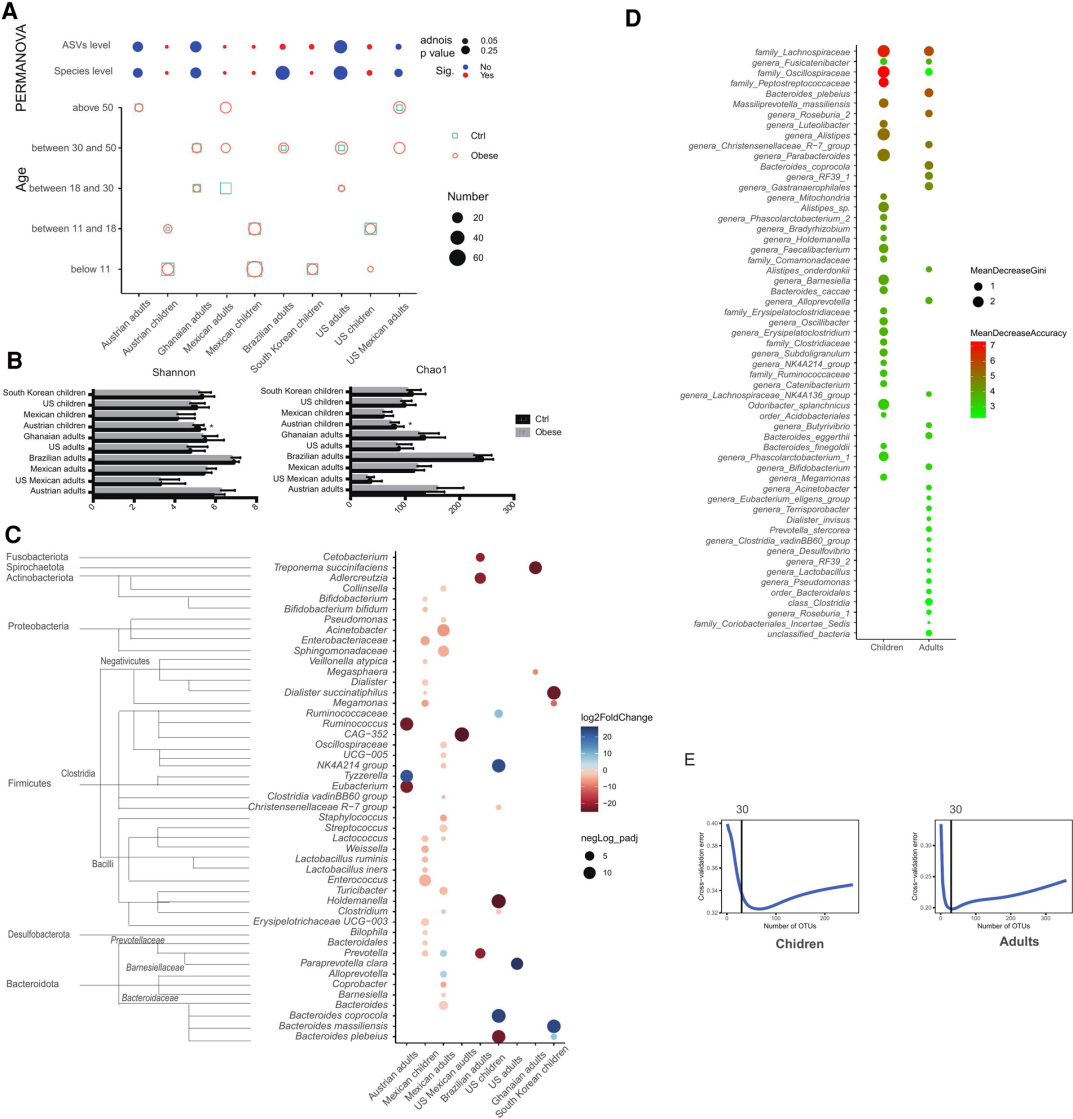
Comparison of gut microbiota between obese and control groups in different cohorts. (**A**) Distance of gut microbiota between obese and control individuals analyzed using PERMANOVA and age distributions in different cohorts; (**B**) Alpha diversity of gut microbiota in different cohort; (**C**) Bacteria with significant difference in abundance between control and obese individuals in different cohorts (Blue means bacteria had higher abundance in control group than obese group, vice versa); (**D**) Top 30 taxa used to predict obesity using the random forest model; (**E**) Cross validation errors of both children cohorts and adults cohorts in random forest model.

Each cohort generally had its unique gut microbiota signature, significantly different from other cohorts (Supplementary Table S12). Therefore, the comparison of gut microbiota between individuals with and without obesity has been performed within a cohort. There was no significant difference in gut bacterial composition between obese and healthy individuals in adult cohorts, except for one study of Mexican adults and ASVs level for the Brazilian study, regardless of the level of ASVs or species (Figure 1A, Supplementary Figures S1,S2). For childhood cohorts, significant differences in gut microbiota were observed between children with and without obesity (Figure 1A, Supplementary Figures S1,S2). To exclude the bias from sample size within a cohort, we used random sampling to match the sample size within the dataset and compared the bacterial composition 100 times. Correspondingly, the percentage of the signs indicating the difference in gut microbiota between health and individuals with obesity was significantly higher in children cohorts than the adult cohorts (Supplementary Table S13). For the Austrian children cohort, the category of BMI was identically sub-grouped in the original study, and we compared the gut microbiota compositions among these subgroups. Children who were overweight, obese and super-obese had significantly different gut microbiota from healthy children and those either at risk or in waste (Supplementary Table S9). Since five of the six adult cohorts only contained females, we re-analyzed children cohorts through gender stratified, except for the Mexican children cohort that did not list the gender information (Supplementary Figures S3). Although the gut microbiota of girls showed differences without significance in two of the three cohorts, the *p*-values were lower than in adult females. Furthermore, the gut microbiota of boys with and without obesity significantly differed. The age distribution is also shown in Figure 1A. Except for Mexican adults, all the studies covered similar age ranges. Alpha diversity index Shannon and Chao1 showed a trend that adulthood gut microbiota was more diverse than childhood ones but was not significant (Figure 1B).

Comparing the difference in abundance of bacteria between obese and non-obese groups, these differences were various depending on cohorts (Figure 1C). More bacteria belonging to Firmicutes and Bacteroidota had a significant difference in abundance, as the increase of Firmicutes and the decrease of Bacteroidota were generally observed in the obese group. Except for the NK4A214 group of the family, *Oscillospiraceae* and *Prevotella* presented different changes between different cohorts; other bacteria had consistent changes along with obesity. For children in Austria, there were no bacteria observed a significant difference in abundance between children with and without obesity.

The random forest model was used for predicting obesity. The correct prediction percentage and prediction error of each cohorts was listed in Supplementary Table S14. In general, children cohorts had higher prediction errors than adults cohorts. Then we combined datasets from these cohorts and performed the prediction using datasets of children and adults separately. Although some samples from adults showed distinct signatures between different cohorts, most samples, especially those from children cohorts, were clustered independently on the population (Supplementary Figure S4, Supplementary Table S15). The general accuracy for the prediction based on merged datasets is minimal, as there would be at least 20% for adults and 32% for children error generated (Supplementary Table S14). As shown in Figure 1D, the top 30 taxa used to predict obesity in children and adults had some overlaps. The family *Lachnospiraceae* is the top1 biomarker for obesity prediction with the highest accuracy.

## Discussion

Childhood obesity is concomitant with the maturation of core gut microbiota and body development; hence it may have a lifelong influence on individuals. Early studies relatively well described the development of gut microbiota in infants and suggested the infant microbiota shaped to a core structure at the age of 3 years (17, 18). However, recent studies revealed that gut microbiota development might take longer, as its evolution and variation can respond to the dietary challenges in school-aged children, yielding new signatures (17, 19, 20). In the present study, we compared the gut microbiota of school-aged children and adults from different cohorts and observed that gut microbiota composition in children altered more intensively due to obesity than in adults. This observation suggests that the gut microbiota in school-aged children has a more vivid response to obesity-related factors than adults, which aligns with previous studies (17). The present study addressed that the gut microbiota composition has no significant difference between adults with and without obesity, which seems different from a previous study on gut microbiota from lean and obese twins (21). However, unlike in children, the difference in core gut microbiota in adults is not at organismal lineage but at the gene level that majorly contributes to the function of gut microbiota (22). Besides the composition of gut microbiota, the lower alpha diversity index was observed in children's gut microbiota than in adults' in the present study, which is in line with the previous reports as the richness of gut microbiota in children increases along with the growth in both first three years and school-ages. Additionally, we observed a higher alpha diversity in the healthy group than the obese group, though only the Austrian children cohort had significance. It suggests that obesity and relevant factors reduced the diversity of gut microbiota. The cohort of US Mexican adults showed a shallow alpha diversity, especially for the Chao1 index. The insufficient sequencing depth introduced a small number of reads (18), leading low alpha diversity index. Altogether, more concerns about gut microbiota in the prevention and intervention of childhood obesity are suggested.

On the taxonomic level, we observed an increase in the ratio of Firmicutes to Bacteroidota in these cohorts in line with previous studies (1, 21). More Firmicutes were found to increase significantly in obese groups and vice versa for Bacteroidota. However, specific bacteria with a significant difference in abundance between individuals with and without obesity depended on cohorts. Commonly used probiotics, such as Bifidobacterium and Lactobacillus, only present significant differences in some cohorts, suggesting they may have minimal impact on others. Therefore, it is challenging to use one golden bacterium to prevent or treat obesity in all cohorts. The family Lachnospiraceae, a propionic acid producer that plays a crucial role in metabolic regulation (22), showed its potential as a promising biomarker and metabolic regulator in the present study. In predication for childhood and adulthood obesity, the relative abundance of the family Lachnospiraceae is the best biomarker.

The present study was technically limited by the raw data generated from different studies and had difficulties in combining all the cohorts to compare the gut microbiota between healthy individuals and those with obesity. Therefore, we discussed the difference within a cohort and summarized common points among different cohorts rather than comparing cohorts horizontally. At both the ASV and assigned taxonomic levels, which helps reduce the bias from sequencing variants, the results are in accordance with the repeated comparison with sample size matched. Moreover, we attempted to elucidate the effects of age and gender. Although part of the cohorts did not display the identical age of the host, we inferred their age range according to the sample size, gender and experimental group in relevant publications. Except for the cohort of Mexican adults, having no overlap of age range between the healthy and obese group, the others had similar age ranges within a cohort, especially in children cohorts. The significant distribution of age causing the significant difference in gut microbiota in Mexican adults reflected the impact of age on the gut microbiota composition, as the previous study (23). Additionally, since the adult cohorts were focused on females and showed no significant difference in gut microbiota between the obese and healthy groups, we stratified the children cohorts. Obesity and relevant factors had less impact on gut microbiota in girls than boys, although girls received more impact than adult females. It suggests gut microbiota is affected by gender, as in previous studies (19, 20), and this effect starts early in childhood. We further performed obesity prediction using random forest model; however, the relevant results only reflect our finding laterally, as the prediction errors are relatively high and combined datasets showed disperse dependent on cohorts to some extent.

In conclusion, the gut microbiota is affected more by obesity and relevant factors in childhood than adulthood. More focus and earlier intervention for childhood obesity are crucial for children's health and lifelong gut microbiota-related health. However, under the limitation of the number and information for released data in the public database, we only included minimal cohorts for this pilot study. We would like to appreciate and encourage more contributions of sequencing data and relevant information for further discovery. Besides, tools with higher accuracy, such as metagenomics and culturomics, should be considered for precise taxonomic classification.

## Data Availability

Publicly available datasets were analyzed in this study, the names of the repositories/accession numbers are provided within the manuscript/**Supplementary Materials**.
