# Supplementary material for: Greater alteration of gut microbiota occurs in childhood obesity than in adulthood obesity
Source: Front Pediatr. 2023 Jan 26;11:1087401. doi: 10.3389/fped.2023.1087401 (PMC9909466; doi:10.3389/fped.2023.1087401)
Supplement: Supplementary file 1 [file Datasheet1.docx]

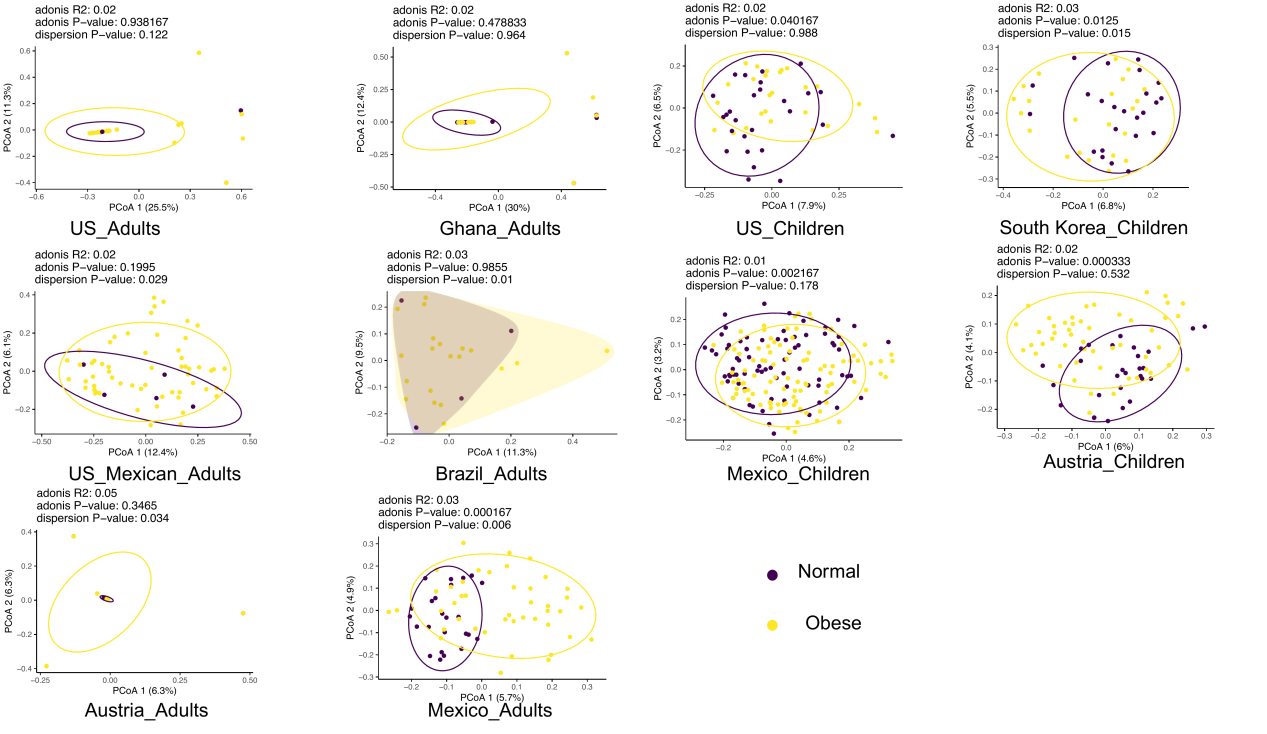


Supplementary Fig 1. PCoA analyses of gut microbiota in different cohorts based on ASVs level.


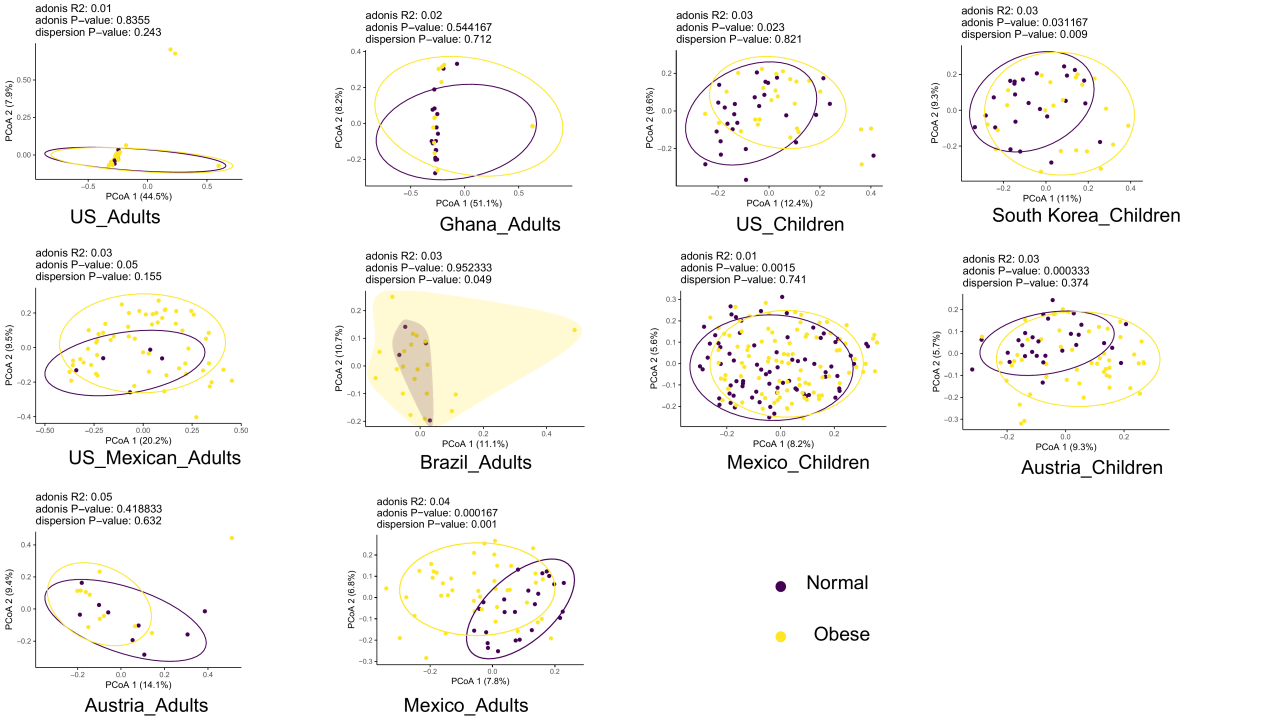


Supplementary Fig 2. PCoA analyses of gut microbiota in different cohorts based on species level.


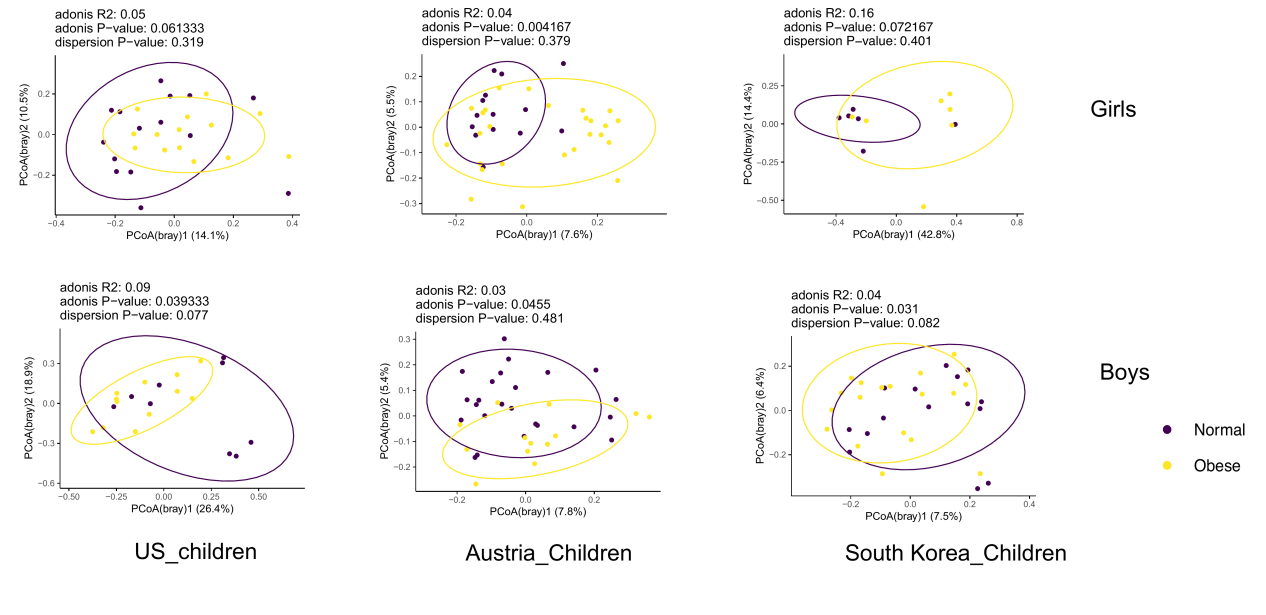


Supplementary Fig 3. PCoA analyses of gut microbiota in different children cohorts, stratified by the gender.


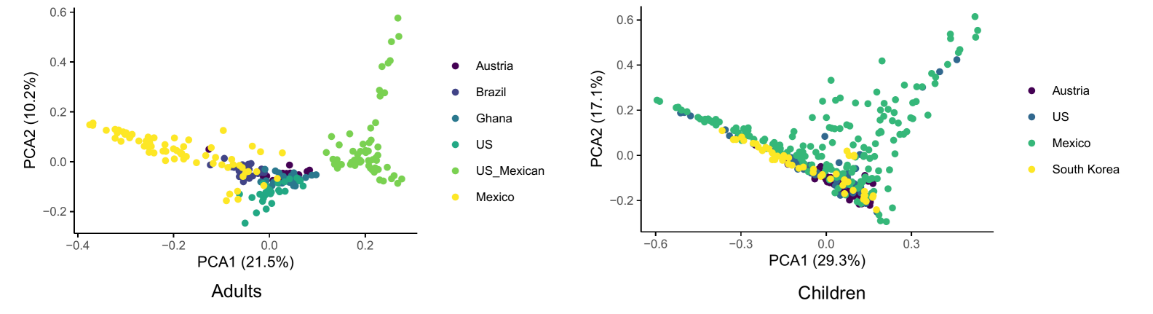


Supplementary Fig 4. PCoA analyses of gut microbiota in combined datasets of adults and children from different cohorts.
